# Supplementary material for: Learning to detect AI texts and learning the limits
Source: PLoS One. 2025 Oct 15;20(10):e0333007. doi: 10.1371/journal.pone.0333007 (PMC12527182; doi:10.1371/journal.pone.0333007)
Supplement: S2 File — All data and scripts used in the study, some additional descriptive statistics, scalable formate figures. (ZIP) [file pone.0333007.s002.tgz › data and scripts/OSF/www/indexStable Universal.htm]

LLM Text Evaluation


# LLM Text Evaluation Experiment

Welcome to our research study. Please proceed to read the informed consent document.

Continue

# Informed Consent

Please read the informed consent document carefully and confirm your participation by checking the box below.

View Informed Consent Document (PDF)

I have read the informed consent document and agree to participate in this study.

Continue

## Instrukce

Pokračovat

# Task 1 out of X

### Text 1

### Text 2

Continue

Který text se vám lépe čte?

Text 1

Text 2

Který text je napsaný člověkem?

Text 1

Text 2

Nakolik jste si svou odpovědí jisti?

Velmi nejistý/á

Velmi jistý/á

OK

## Thank you for participating!

You've completed all the tasks. Click the button below to send your answers and see your results.

Send answers and show my results

Close Review
